# Autosomal dominant variants in *FOXJ1* causing primary ciliary dyskinesia in two patients with obstructive hydrocephalus

**DOI:** 10.1002/mgg3.1726

**Published:** 2021-06-15

**Authors:** Adam J. Shapiro, Kimberley Kaspy, M. Leigh Ann Daniels, Jaclyn R. Stonebraker, Van‐Hung Nguyen, Lyne Joyal, Michael R. Knowles, Maimoona A. Zariwala

**Affiliations:** ^1^ Division of Pediatric Respiratory Medicine McGill University Health Centre Research Institute Montreal QC Canada; ^2^ Division of Pulmonary Diseases and Critical Care Medicine Department of Medicine University of North Carolina at Chapel Hill Chapel Hill NC USA; ^3^ School of Medicine Marsico Lung Institute University of North Carolina at Chapel Hill Chapel Hill NC USA; ^4^ Department of Pathology McGill University Health Centre Montreal QC Canada; ^5^ Department of Medicine Marsico Lung Institute University of North Carolina at Chapel Hill Chapel Hill NC USA; ^6^ Department of Pathology and Laboratory Medicine Marsico Lung Institute University of North Carolina at Chapel Hill Chapel Hill NC USA

## Abstract

**Background:**

Primary ciliary dyskinesia (PCD) is a mostly autosomal recessive, genetic disease of abnormal motile cilia function, resulting in bronchiectasis, infertility, organ laterality defects, and chronic otolaryngology disease. Though motile, ependymal cilia influencing cerebrospinal fluid flow in the central nervous system share many aspects of structure and function with motile cilia in the respiratory tract, hydrocephalus is rarely associated with PCD. Recently, pathogenic variants in *FOXJ1* (Chr 17q25.1) were identified causing PCD associated with hydrocephalus, reduced respiratory cilia number, axonemal microtubule disorganization, and occurring in a *de novo*, autosomal dominant inheritance pattern.

**Method:**

Two patients with chronic oto‐sino‐pulmonary disease and hydrocephalus underwent candidate testing of *FOXJ1*. Coding region and splice junctions were sequenced and analyzed under the auspices of Genetic Disorders of Mucociliary Clearance Consortium.

**Results:**

Upon sequencing of the entire coding region and splice‐junctions, heterozygous, pathogenic variants in *FOXJ1* were discovered in exon 3 of two patients: an 11‐month‐old female with situs inversus totalis (NM_001454.4: c.945delC (p.Phe315Leufs*18)) and a 51 year‐old male, post‐double lung transplantation (NM_001454.4: c.929_932delACTG (p.Asp310Glyfs*22)). *FOXJ1* variants were not detected in the available parents and the siblings of these probands.

**Conclusion:**

*FOXJ1* pathogenic variants cause PCD in a *de novo*, autosomal dominant inheritance pattern, and are associated with hydrocephalus. Physicians treating patients with hydrocephalus and chronic oto‐sino‐pulmonary disease should be aware of this PCD association and test for *FOXJ1* variants.

## Graphical abstract

Variants in FOXJ1 are a rare cause of respiratory ciliary dysfunction and hydrocephalus, but few cases have been previously illustrated. We report 2 cases of primary ciliary dyskinesia (PCD) with hydrocephalus, both caused by *de novo*, autosomal dominant, variants in FOXJ1. FOXJ1 should be examined in patients with hydrocephalus and classic PCD symptoms of chronic oto‐sino‐pulmonary disease, neonatal respiratory distress, and organ laterality defects.

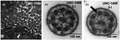

## 1 INTRODUCTION

Primary ciliary dyskinesia (PCD, OMIM 244400) is a rare inherited disease of abnormal mucociliary clearance in the upper and lower airways, resulting in bronchiectasis, recurrent lower respiratory tract infections, infertility, organ laterality defects, and chronic otolaryngology disease. To date, over 50 genes have been associated with PCD, and autosomal recessive inheritance patterns predominate in this respiratory disease, aside from a few rare, X‐linked genes (Zariwala et al., 1993). In 2019, Wallmeier and colleagues proposed that heterozygous, pathogenic *FOXJ1* (OMIM 618699, 602291) variants in exon 3, inherited in *de novo*, autosomal dominant patterns, cause motile, respiratory ciliary disease (Wallmeier et al., 2019). These variants result in obstructive hydrocephalus internus and a motile ciliopathy with markedly decreased respiratory cilia number per epithelial cell, including frequent microtubule disorganization of ciliary cross sections. Though diagnosed as reduced generation of multiple motile cilia (RGMC), the patients in that series showed clinical phenotypes consistent with PCD, including organ situs anomalies, neonatal respiratory distress, chronic cough and sino‐nasal disease from infancy, bronchiectasis, and abnormal ciliary waveform patterns on high‐speed video microscopy.

In this report, we describe two individuals with obstructive hydrocephalus internus and classic PCD clinical phenotypes who underwent targeted sequencing of *FOXJ1*. Heterozygous, pathogenic/likely pathogenic variants were identified in exon 3 of *FOXJ1* for both subjects, and inheritance of these variants was confirmed as *de novo* upon segregation analysis. Contrary to past *FOXJ1* analyses (Wallmeier et al., 2019), transmission electron microscopy showed apparent normal numbers of ciliary axonemes and apically arranged ciliary basal bodies on respiratory epithelial cells from nasal scrape samples. Further ciliary ultrastructural analysis revealed normal 9 + 2 arrangement on nearly all ciliary cross sections and only minimal evidence of microtubule disorganization.

## 2 MATERIALS AND METHODS

Both participants and the available family members provided informed consent for research studies. All protocols, including the Genetic Disorders of Mucociliary Clearance Consortium (GDMCC), were approved through the institutional review board at the University of North Carolina at Chapel Hill. Two unrelated patients in this study had hydrocephalus as well as a clinical phenotype consistent with PCD (Shapiro et al., 2016, 2018; Zariwala et al., 1993). Nasal nitric oxide was measured with an Ecophysics (Durnten, Switzerland) CLD88 chemiluminescence analyzer per standardized clinical protocol (Shapiro et al., 2020). Ciliary ultrastructure was assessed on epithelial cells obtained noninvasively by scraping the inferior nasal turbinate technique (Carson & Collier, 1988). Nasal epithelial cells were immediately fixed in 2.5% glutaraldehyde in 0.1 M phosphate buffer and post‐fixed in 1% osmium tetroxide (OsO4). Once properly fixed, the sample was dehydrated and embedded in propylene oxide/EPON™ mix. Ultrathin sections were stained with uranyl acetate followed by lead citrate and observed with a transmission electron microscope (Carl Zeiss) operating at 60 kV. Greater than 50 focused images of various axonemal cross sections throughout the tissue sample were assessed as a complete ultrastructural study.

### 2.1 Genetic analysis

Both coding exons 2 and 3 and the splice junctions of *FOXJ1* (NM_001454.4) were amplified for probands as well as available family members using M13 tagged gene‐specific primers from the flanking introns. Amplification primers for exon 2 were 5′‐M13‐GTACACACACTGTCCCCTCG‐3′ (forward) and 5′‐M13‐TTGGCCTGCAGATTTGGGAT‐3′ (reverse) and for exon 3 were 5′‐M13‐CTCTTGCTCTCTCCCTGCAC‐3′ (forward) and 5′‐M13‐GACTTGGGCACTGTCCAGAG‐3′ (reverse). Polymerase chain reaction (PCR) was carried out using 20–100 ng of genomic DNA, 400 pmol of each primer, 1X PCR buffer+1.5 mM MgCl_2_ (Applied Biosystem™), 0.2 mM total dNTP (Promega™), 7.5% dimethyl sulfoxide (Sigma‐Aldrich), and 0.05 units of AmpliTaq polymerase (Sigma‐Aldrich). Cycle conditions (ABI 2720 Thermal Cycler, Applied Biosystem™) included initial denaturation at 94℃ for 5 min, followed by 35 cycles of denaturation at 94℃ for 30 s, annealing at 60℃ for 45 s, and extension at 72℃ for 45 s, followed by a final extension at 72℃ for 10 min. Amplified products were visualized by 1% agarose gel electrophoresis, and treated with ExoSAP‐IT (Applied Biosystem™) prior to carrying out Sanger sequencing using M13 primers.

## 3 RESULTS

### 3.1 Case 1

**FIGURE 1:**
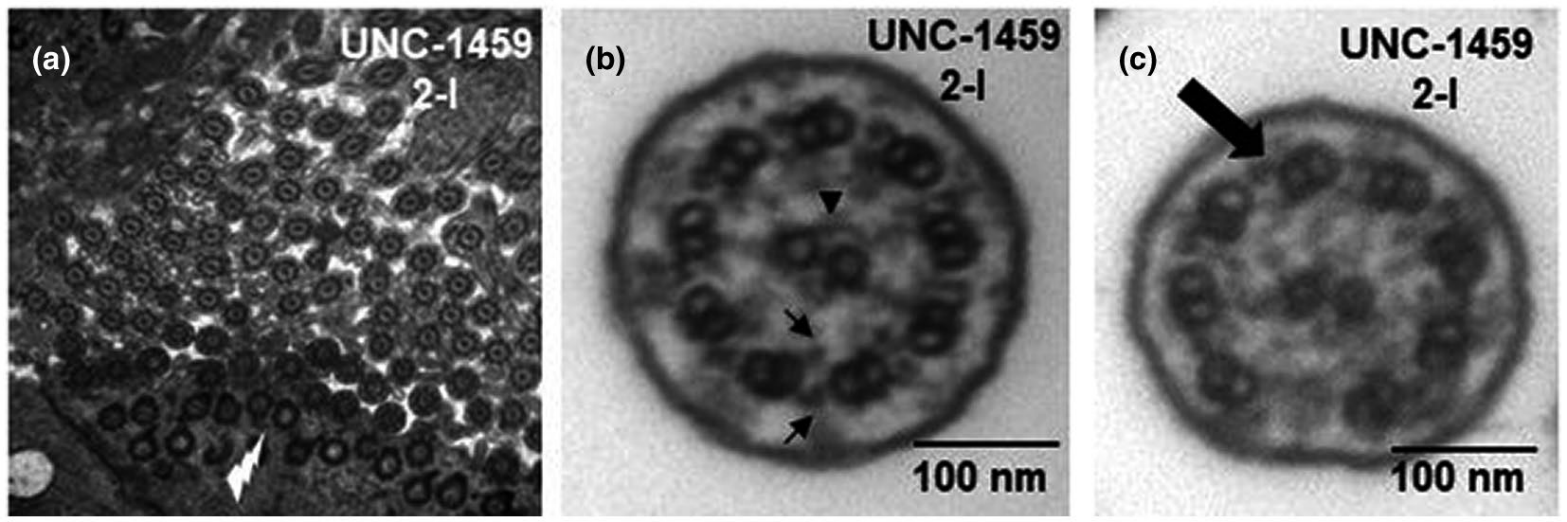
Ciliary electron microscopy images in PCD proband. (a) Low power view (30,000×) of nasal cilia on transmission electron microscopy from proband 2‐1 (UNC‐1459). Note the normally arranged, apical basal bodies (white flash) and numerous ciliary axonemal cross sections per this single nasal epithelial cell. (b) Transmission electron micrograph of an axonemal cross section of nasal epithelium from a proband 2‐1 (UNC‐1459) demonstrating normal ciliary ultrastructure with the characteristic nine outer microtubule doublets surrounding the central pair (filled triangle). Outer and inner dynein arms are projected from each outer doublet (arrows). (c) Example of microtubule disorganization (8 + 2 transposition defect) seen on only 4 individual cross sections from proband 2‐1 (UNC‐1459), with normal outer dynein arms (black arrow)

A healthy infant female (UNC‐1459 2‐I) of Ashkenazi Jewish heritage, born at 42 weeks gestational age, required 12 post‐natal days in the hospital for neonatal respiratory distress and supplemental oxygen therapy. Postnatal chest radiography, echocardiography, and abdominal ultrasonography confirmed situs inversus totalis with a small atrial septal defect. A commercial panel of 35 PCD‐associated genes did not reveal any variants of interest, and nasal ciliary biopsy for electron microscopy showed abundant cilia per epithelial cell (>30/cell at 30,000× magnification) with normally aligned, apical basal bodies. Ciliary ultrastructure was largely unremarkable, with over 1000 individual cross sections showing normal 9 + 2 microtubule arrangement, the clear presence of the central pairs, normal inner and outer dynein arms, and only five cross sections (<1%) with evidence of microtubule disorganization anomalies (Figure 1).

With daily nasal congestion and wet cough from the first week of life, the child was clinically followed per PCD Foundation consensus statement recommendations (Shapiro et al., 2016), treated with daily inhaled hypertonic saline plus chest physiotherapy, and repeat pharyngeal swab cultures grew methicillin‐resistant *Staphylococcus aureus*, *Haemophilus influenzae*, and *Streptococcus* *agalactiae*. She did not manifest recurrent pneumonias or respiratory exacerbations. At 3 months of age, she developed lethargy and poor feeding associated with an Influenza B infection. Concurrently, her head circumference acutely increased from 34 cm (50%) at birth to 42 cm (99%), and an urgent MRI brain scan revealed stenosis of the distal aqueduct of Sylvius with severe obstructive supratentorial hydrocephalus. Ventriculoperitoneal shunting was urgently performed with good success.

**FIGURE 2:**
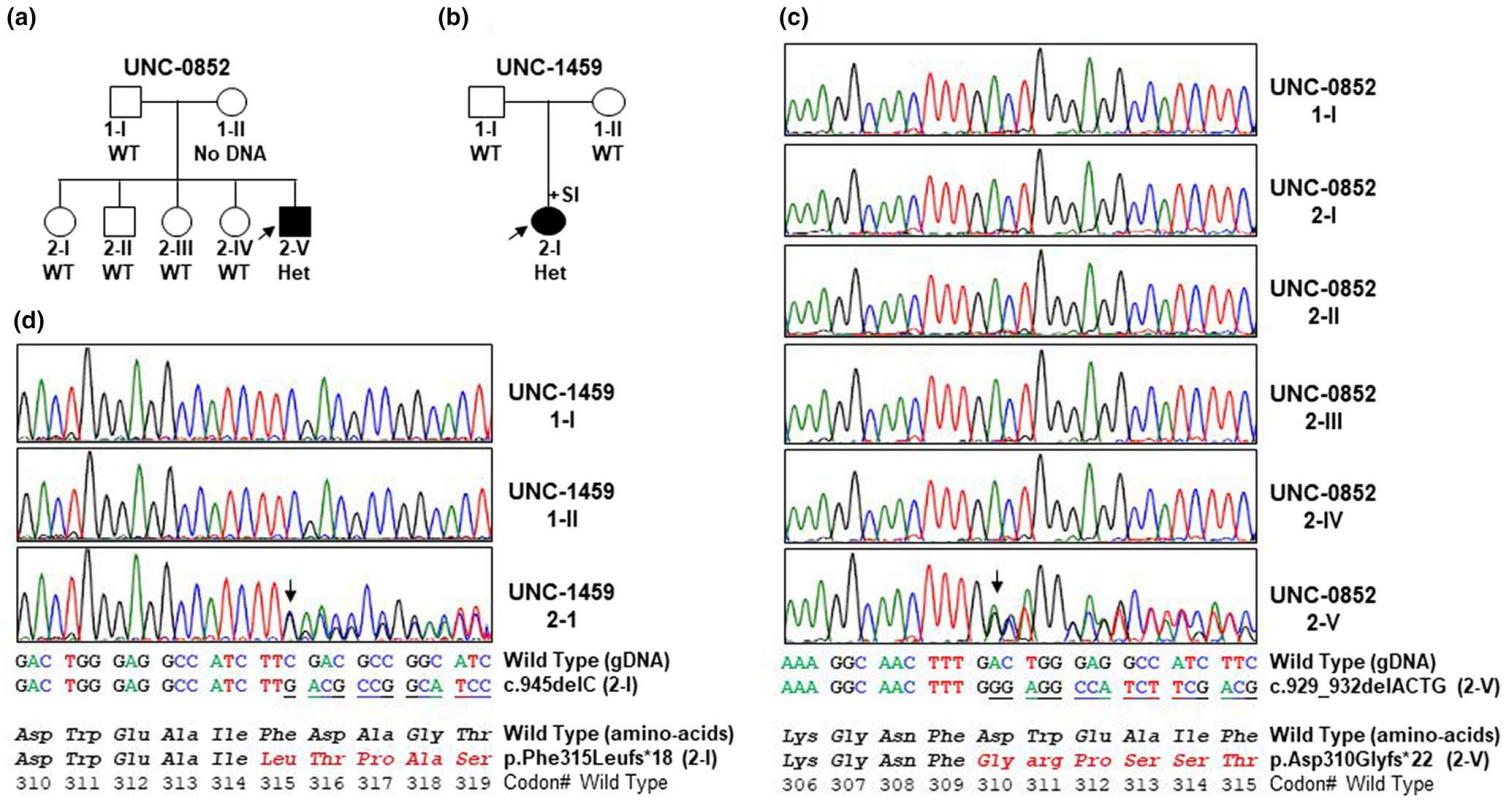
*FOXJ1* (NM_001454.4) variants in PCD subjects. (a and b) Pedigrees showing the presence of heterozygous de novo pathogenic variant in *FOXJ1* (NM_001454.4; Chr.17q25.1) in the affected individuals, consistent with an autosomal dominant trait. Males and female genders are designated by squares and circles, respectively. Filled symbols indicate PCD affected individuals. Probands are designated by an arrow. WT (wild type reference genomic DNA sequence), Het (heterozygous pathogenic variant), and +SI (presence of situs inversus) are indicated. (c and d) Electropherograms of Sanger sequencing of exon 3 for families UNC‐0852 (c) and UNC‐1459 (d). None of the available unaffected parents or siblings harbored pathogenic variant, consistent with de novo autosomal dominant trait. Base sequence, amino‐acid sequence and codon numbers are shown. Position of c.945delC and c.929_932delACTG pathogenic alleles are shown by an arrow. Base sequence is underlined and amino‐acids are designated with red fonts to indicate the position of pathogenic variants

The seven siblings of the proband were clinically evaluated for PCD, and aside from recurrent otitis media requiring ventilation tube placement in 3 siblings, all lacked a PCD phenotype or hydrocephalus, and nasal nitric oxide levels were normal. Thus, genetic analysis of *FOXJ1* was not pursued in the siblings. There was no reported parental consanguinity. With these results, a *de novo* genetic variant was suspected, and targeted research sequencing of the *FOXJ1* gene (NM_001454.4), residing on chromosome 17q25.1, was performed in the proband and her parents. A heterozygous, frameshift, pathogenic variant was found upon full coding region sequencing in the proband at c.945delC (p.Phe315Leufs*18) in exon 3. This variant was not identified in either parent (Figure 2).

At 11 months of life, the proband and her parents developed acute viral symptoms and positive PCR testing for the SARS‐CoV‐2 virus. Remarkably, symptoms were limited to mild cough and fever for 48 hr without emergency care or other lasting sequelae of infection. At 1 year of age, she has not developed recurrent otitis media but did require adenoidectomy for severe obstructive sleep apnea.

### 3.2 Case 2

A 51‐year‐old male (UNC‐0852 2‐V) was investigated for possible PCD after undergoing double lung transplantation at age 43. Sweat chloride testing, *CFTR* genotyping, and immunology labs were normal. Ciliary electron microscopy was not performed in this case, and the patient had a normal nasal nitric oxide level at 234 nL/min. Prior to transplantation, he had year‐round productive cough since early infancy, two hospitalizations annually for acute respiratory exacerbations, and diffuse bronchiectasis that was first discovered at 26 years of age. His sputum cultures routinely grew *Staphylococcus aureus* and Aspergillus species but were eventually colonized with *Pseudomonas aeruginosa*, and lung function pre‐transplantation was quite impaired, with an FEV1 of 0.84L (23% predicted).

Despite a term birth, the patient had neonatal respiratory distress as well as recurrent otitis media with speech delay in childhood, though he never required tympanostomy tubes. He endorsed year‐round (non‐seasonal) nasal congestion from the first year of life, with chronic sinusitis and nasal polyposis evident on the CT scan of the sinuses. There were no identified organ laterality defects upon CT or ultrasound imaging of the heart, thorax, or abdomen. His physical examination was notable for diffuse, low‐pitched wheezing and digital clubbing. Further surgical history was remarkable for ventriculoperotineal shunt placement for hydrocephalus discovered shortly after birth (with multiple subsequent revisions), left lower lobectomy at 4 years old, and scoliosis correction in adolescence. Family history revealed the patient was unmarried and never had children. He was of mixed Irish (father) and Irish/Native American (mother) heritage, and there were no similarly affected siblings or extended family members. Mutation profiling of the full coding region revealed a frameshift, heterozygous pathogenic variant at c.929_932delACTG (p.Asp310Glyfs*22) in exon 3 of the *FOXJ1* gene (NM_001454.4). The proband's father and four unaffected siblings did not carry this variant. Unfortunately, genetic material for maternal testing was not available (Figure 2).

## 4 DISCUSSION

Limited information is currently known about the role of *FOXJ1* in respiratory cilia dysfunction or hydrocephalus in humans, and it is likely quite rare as a cause of PCD. Until recently, candidate gene testing of small numbers of subjects with clinically diagnosed PCD or hydrocephalus plus organ laterality defects did not reveal any cases associated with *FOXJ1* variants (Kosaki et al., 2004; Maiti et al., 2000). Early studies in animal models demonstrated *FOXJ1* variants result in a motile ciliopathy with reduced ciliary numbers and mislocalized basal bodies in ciliated cells of the brain, airways, and embryonic left‐right nodal organizer (Brody et al., 2000; Hackett et al., 1995; Stubbs et al., 2008; Wallmeier et al., 2019). Congruently, our cases exhibit classic respiratory symptoms associated with the motile cilia dysfunction seen in PCD (Leigh et al., 2016), hydrocephalus internus appearing in infancy, and an organ laterality defect in one case. However, electron microscopy, in this case, demonstrates normal respiratory cilia number per nasal epithelial cell, normally aligned apical basal bodies, and normal ciliary ultrastructure in the vast majority of examined axonemal cross sections. This distinct difference from the previous reports (Wallmeier et al., 2019) means that respiratory cilia production in patients with *FOXJ1* mutations can be variable, not always consistent with a diagnosis of RGMC, and this gene should not be discounted as a potential cause of PCD based upon normal transmission electron microscopy images. Additionally, with normal nasal nitric oxide levels seen in our second case, nasal nitric oxide measurement, which is commonly used as a PCD screening test, cannot be used to reliably screen for PCD from *FOXJ1* variants. Lastly, though we did not perform immunofluorescence testing for detection of ciliary axonemal proteins, past analysis has also shown that immunofluorescence testing is normal in PCD caused by *FOXJ1* variants, thus limiting the diagnosis of these cases to mainly genetic testing (Wallmeier et al., 2019).

Previously reported *FOXJ1* cases resulting in RGMC were all associated with premature termination codons (nonsense or frameshift alleles) in the *FOXJ1* C‐terminal region at a small hotspot on exon 3 (Wallmeier et al., 2019; Table 1). Similar to those reports, our cases have their *FOXJ1* pathogenic variant in the same hotspot region on exon 3 predicted to result in loss of protein function. Thus, the normal ciliary number and ultrastructure in our first case do not seem explained by the location or type of *FOXJ1* variant. Recently, a large genetic study on congenital hydrocephalus reported 3 unrelated cases harboring heterozygous variants in *FOXJ1* (Jin et al., 2020). Authors report that none of their cases had cardiac or pulmonary symptoms (Table 1), however, they do not provide the ages or full clinical phenotypes of the patients, and hence it is difficult to ascertain if their cases had subtle symptoms of PCD. Two of their pedigrees showed *de novo*, loss of function variants in the same hot spot region of exon 3. However, in their third case, a missense variant was residing in exon 2 and was inherited from the mother who did not appear to harbor hydrocephalus. This case argues that female fertility may not be affected; however, the pathogenicity of this missense variant is uncertain because no functional studies were performed. Thus, the effect of *FOXJ1* pathogenic variants on fertility is unclear, though severe oligoasthenoteratospermia was previously reported in US‐2 II1 (Wallmeier et al., 2019; Table 1). It is interesting to note that in this small series of cases, two variants (c.967delG and c.826C>T) have occurred in more than one unrelated family, albeit arising *de novo*, again suggesting the presence of a hot spot.

**TABLE 1:** Summary of pathogenic variants in *FOXJ1* (NM_001454.4), previously published, and this current report

Case no.	*FOXJ1* variants (exon no.; base change, protein change)	Gender	Inheritance	Obstructive hydrocephalus	Disease status
OP1743 II1 (Wallmeier et al., 2019)	Exon 3; c.901G>T; p Glu310*	Male	AD, *de novo*	Yes	PCD
OP1933 II1 (Wallmeier et al., 2019)	Exon 3; c.826C>T; p.Gln276*	Male	AD, *de novo*	Yes	PCD + VSD
OP2950 II1 (Wallmeier et al., 2019)	Exon 3; c.868_871dupACGA; p.Thr291Lysfs*12	Female	AD, *de* *novo*	Yes	PCD + SI
RBH II1 (Wallmeier et al., 2019)	Exon 3; c.967delG; p.Glu323Serfs*10	Female	AD, *de novo*	Yes	PCD
US‐1 II1 (Wallmeier et al., 2019)	Exon 3; c.826C>T; p.Gln276*	Male	AD, *de novo*	Yes	PCD + SI
US‐2 II1 (Wallmeier et al., 2019)	Exon 3; c.939delC; p.Ile314Serfs*19	Male	AD, *de novo*?	Yes	PCD + SI
KCHYD109‐1 (Jin et al., 2020)	Exon 2; c.287C>G; p.Thr96Arg	Male	AD, carrier mother^a^	Yes	Absent cardiac & pulmonary symptoms
KCHYD376‐1 (Jin et al., 2020)	exon 3; c.826C>T; p.Gln276*	Male	AD, *de novo*	Yes	Absent cardiac & pulmonary symptoms
KCHYD238‐4 (Jin et al., 2020)	exon 3; c.967delG; p.Glu323Serfs*10	Female	AD, *de novo*	Yes	Absent cardiac & pulmonary symptoms
UNC‐0852 (current report)	Exon 3; c.945delC; p.Phe315Leufs*18	Male	AD, *de novo*? (father & 3 sibs are wild type)	Yes	PCD
UNC‐1459 (current report)	Exon 3; c.929_932delACTG; p.Asp310Glyfs*22	Female	AD, de novo	Yes	PCD + SI/ASD

Abbreviations: AD, autosomal dominant; ASD, atrial septal defect; PCD, primary ciliary dyskinesia; SI, situs inversus totalis; VSD, ventricular septal defect.

^a^ No clinical information on mother's status. Missense variant thus appears to be a variant of uncertain significance.

Some *FOXJ1* cases may lack hydrocephalus from birth, as the onset of hydrocephalus seen with *FOXJ1* can be delayed for months and even occur in adulthood (Wallmeier et al., 2019). This is likely due to ongoing *FOXJ1*‐related ependymal cell maturation occurring post‐natally, but the mechanisms of this are poorly understood (Vidovic et al., 2018). It is interesting that our first case only developed clinically detectable hydrocephalus at the same time as an Influenza infection, casing us to hypothesize a second insult overwhelmed her prior, intact capacity for adequate flow of cerebrospinal fluid despite her *FOXJ1* variant. Further exploration of ciliary protein involvement in normal human cerebrospinal flow is necessary to explain this delayed onset of hydrocephalus in *FOXJ1* associated cases.

*MCIDAS* and *CCNO* are two essential genes in the early, NOTCH1‐dependant signaling pathway that interact with *FOXJ1* for normal ciliogenesis and multiciliated cell formation (Lewis & Stracker, 2020). Pathogenic variants in *CCNO* and *MCIDAS* result in RGMC, hydrocephalus in some affected individuals, and severe respiratory phenotypes in some patients, with rapid deterioration in lung function reported (Amirav et al., 2016). However, similar severe phenotypes have not been reported with *FOXJ1* in humans. Interestingly, the clinical respiratory phenotypes in our cases range from mild (no respiratory exacerbations in the first year of life and well‐tolerated SARS‐CoV‐2 infection) to severe and life‐threatening (respiratory failure requiring lung transplantation in the fourth decade of life). While these outcomes may be a reflection of advancing age and environmental insults rather than strictly per genotype, further analysis of long‐term respiratory outcomes in *FOXJ1* patients is necessary to know if this gene acts as severely as other genes implicated in the human ciliogenesis pathway.

In conclusion, *FOXJ1* should be examined in patients with hydrocephalus and classic PCD symptoms of chronic oto‐sino‐pulmonary disease, neonatal respiratory distress, and organ laterality defects. Identification of more patients with *FOXJ1*‐related respiratory ciliopathies will expand the known phenotype and provide valuable prognostic data to guide future medical therapies in these cases.

## CONFLICT OF INTEREST

The authors declare no conflict of interest.

## AUTHOR CONTRIBUTIONS

All authors were directly involved in data collection and analysis and in manuscript creation plus editing.

## Data Availability

The data that support the findings of this study are available from the corresponding author upon reasonable request.

## References

[mgg31726-bib-0001] Amirav, I., Wallmeier, J., Loges, N. T., Menchen, T., Pennekamp, P., Mussaffi, H., Abitbul, R., Avital, A., Bentur, L., Dougherty, G. W., & Nael, E. (2016). Systematic analysis of CCNO variants in a defined population: implications for clinical phenotype and differential diagnosis. Human Mutation, 37(4), 396–405. 26777464 10.1002/humu.22957

[mgg31726-bib-0002] Brody, S. L., Yan, X. H., Wuerffel, M. K., Song, S. K., & Shapiro, S. D. (2000). Ciliogenesis and left‐right axis defects in forkhead factor HFH‐4‐null mice. American Journal of Respiratory Cell and Molecular Biology, 23(1), 45–51. 10.1165/ajrcmb.23.1.4070 10873152

[mgg31726-bib-0003] Carson, J. L., & Collier, A. M. (1988). Ciliary defects: Cell biology and clinical perspectives. Advances in Pediatrics, 35, 139–165. 3055856

[mgg31726-bib-0004] Hackett, B. P., Brody, S. L., Liang, M., Zeitz, I. D., Bruns, L. A., & Gitlin, J. D. (1995). Primary structure of hepatocyte nuclear factor/forkhead homologue 4 and characterization of gene expression in the developing respiratory and reproductive epithelium. Proceedings of the National Academy of Sciences of the United States of America, 92(10), 4249–4253. 10.1073/pnas.92.10.4249 7753791 PMC41921

[mgg31726-bib-0005] Jin, S. C., Dong, W., Kundishora, A. J., Panchagnula, S., Moreno‐De‐Luca, A., Furey, C. G., Allocco, A. A., Walker, R. L., Nelson‐Williams, C., Smith, H., Dunbar, A., Conine, S., Lu, Q., Zeng, X., Sierant, M. C., Knight, J. R., Sullivan, W., Duy, P. Q., DeSpenza, T., … Kahle, K. T. (2020). Exome sequencing implicates genetic disruption of prenatal neuro‐gliogenesis in sporadic congenital hydrocephalus. Nature Medicine, 26(11), 1754–1765. 10.1038/s41591-020-1090-2 PMC7871900 33077954

[mgg31726-bib-0006] Kosaki, K., Ikeda, K., Miyakoshi, K., Ueno, M., Kosaki, R., Takahashi, D., Tanaka, M., Torikata, C., Yoshimura, Y., & Takahashi, T. (2004). Absent inner dynein arms in a fetus with familial hydrocephalus‐situs abnormality. American Journal of Medical Genetics, 129A(3), 308–311. 10.1002/ajmg.a.30177 15326634

[mgg31726-bib-0007] Leigh, M. W., Ferkol, T. W., Davis, S. D., Lee, H.‐S., Rosenfeld, M., Dell, S. D., Sagel, S. D., Milla, C., Olivier, K. N., Sullivan, K. M., Zariwala, M. A., Pittman, J. E., Shapiro, A. J., Carson, J. L., Krischer, J., Hazucha, M. J., & Knowles, M. R. (2016). Clinical features and associated likelihood of primary ciliary dyskinesia in children and adolescents. Annals of the American Thoracic Society, 13(8), 1305–1313. 10.1513/AnnalsATS.201511-748OC 27070726 PMC5021075

[mgg31726-bib-0008] Lewis, M., & Stracker, T. H. (2020). Transcriptional regulation of multiciliated cell differentiation. Seminars in Cell & Developmental Biology, 110, 51–60. 10.1016/j.semcdb.2020.04.007 32362381

[mgg31726-bib-0009] Maiti, A. K., Bartoloni, L., Mitchison, H. M., Meeks, M., Chung, E., Spiden, S., Gehrig, C., Rossier, C., DeLozier‐Blanchet, C. D., Blouin, J.‐L., Gardiner, R. M., & Antonarakis, S. E. (2000). No deleterious mutations in the FOXJ1 (alias HFH‐4) gene in patients with primary ciliary dyskinesia (PCD). Cytogenetic and Genome Research, 90(1‐2), 119–122. 10.1159/000015645 11060460

[mgg31726-bib-0010] Shapiro, A. J., Davis, S. D., Polineni, D., Manion, M., Rosenfeld, M., Dell, S. D., Chilvers, M. A., Ferkol, T. W., Zariwala, M. A., Sagel, S. D., & Josephson, M. (2018). Diagnosis of primary ciliary dyskinesia. An official American thoracic society clinical practice guideline. American Journal of Respiratory and Critical Care Medicine, 197(12), e24–e39. 29905515 10.1164/rccm.201805-0819ST PMC6006411

[mgg31726-bib-0011] Shapiro, A. J., Dell, S. D., Gaston, B., O’Connor, M., Marozkina, N., Manion, M., Hazucha, M. J., & Leigh, M. W. (2020). Nasal nitric oxide measurement in primary ciliary dyskinesia. A technical paper on standardized testing protocols. Annals of the American Thoracic Society, 17(2), e1–e12. 10.1513/AnnalsATS.201904-347OT 31770003 PMC6993796

[mgg31726-bib-0012] Shapiro, A. J., Zariwala, M. A., Ferkol, T., Davis, S. D., Sagel, S. D., Dell, S. D., Rosenfeld, M., Olivier, K. N., Milla, C., Daniel, S. J., Kimple, A. J., Manion, M., Knowles, M. R., & Leigh, M. W. (2016). Diagnosis, monitoring, and treatment of primary ciliary dyskinesia: PCD foundation consensus recommendations based on state of the art review. Pediatric Pulmonology, 51(2), 115–132. 10.1002/ppul.23304 26418604 PMC4912005

[mgg31726-bib-0013] Stubbs, J. L., Oishi, I., Izpisúa Belmonte, J. C., & Kintner, C. (2008). The forkhead protein Foxj1 specifies node‐like cilia in Xenopus and zebrafish embryos. Nature Genetics, 40(12), 1454–1460. 10.1038/ng.267. 19011629 PMC4648715

[mgg31726-bib-0014] Vidovic, D., Davila, R. A., Gronostajski, R. M., Harvey, T. J., & Piper, M. (2018). Transcriptional regulation of ependymal cell maturation within the postnatal brain. Neural Development, 13(1), 2. 10.1186/s13064-018-0099-4 29452604 PMC5816376

[mgg31726-bib-0015] Wallmeier, J., Frank, D., Shoemark, A., Nöthe‐Menchen, T., Cindric, S., Olbrich, H., Loges, N. T., Aprea, I., Dougherty, G. W., Pennekamp, P., Kaiser, T., Mitchison, H. M., Hogg, C., Carr, S. B., Zariwala, M. A., Ferkol, T., Leigh, M. W., Davis, S. D., Atkinson, J., … Omran, H. (2019). De novo mutations in FOXJ1 result in a motile ciliopathy with hydrocephalus and randomization of left/right body asymmetry. The American Journal of Human Genetics, 105(5), 1030–1039. 10.1016/j.ajhg.2019.09.022 31630787 PMC6849114

[mgg31726-bib-0016] Zariwala, M. A., Knowles, M. R., & Leigh, M. W. (1993). Primary ciliary dyskinesia. In M. P. Adam, H. H. Ardinger, & R. A. Pagon et al., GeneReviews(®). University of Washington. 20301301

